# An Approach to Developing Local Climate Change Environmental Public Health Indicators in a Rural District

**DOI:** 10.1155/2017/3407325

**Published:** 2017-03-02

**Authors:** Adele Houghton, Jessica Austin, Abby Beerman, Clayton Horton

**Affiliations:** ^1^Biositu, LLC, 505D W Alabama St., Houston, TX 77006, USA; ^2^Green River District Health Department, 1501 Breckenridge Street, Owensboro, KY 42303, USA

## Abstract

Climate change represents a significant and growing threat to population health. Rural areas face unique challenges, such as high rates of vulnerable populations; economic uncertainty due to their reliance on industries that are vulnerable to climate change; less resilient infrastructure; and lower levels of access to community and emergency services than urban areas. This article fills a gap in public health practice by developing climate and health environmental public health indicators for a local public health department in a rural area. We adapted the National Environmental Public Health Tracking Network's framework for climate and health indicators to a seven-county health department in Western Kentucky. Using a three-step review process, we identified primary climate-related environmental public health hazards for the region (extreme heat, drought, and flooding) and a suite of related exposure, health outcome, population vulnerability, and environmental vulnerability indicators. Indicators that performed more poorly at the county level than at the state and national level were defined as “high vulnerability.” Six to eight high vulnerability indicators were identified for each county. The local health department plans to use the results to enhance three key areas of existing services: epidemiology, public health preparedness, and community health assessment.

## 1. Introduction

Climate change represents a significant and growing threat to population health [1]. Health effects take the form of exacerbating existing, known hazards—such as increasing the risk of morbidity and mortality during heat waves [2]—and introducing novel health risks—such as Harmful Algal Blooms (HABs) erupting for the first time in warming waters [3].

Rural areas, comprising 95% of US landmass but housing only 19% of the population [4, p. 334], are particularly vulnerable to the health effects of climate change ([4, p. 339]; [5, 6]). Natural climatic processes, such as airflow, often manifest differently in rural areas versus their urban counterparts. For example, ground level ozone, a key component of smog, is often associated with urban areas because motor vehicle exhaust is a major emitter of ozone precursors such as nitrogen oxide (NO*x*) [7]. However, rural areas can also experience high concentrations of ground level ozone when air currents transport the gas from the stratosphere or upper troposphere to the ground [8]. The meteorological shifts caused by climate change are likely to increase ozone events in both urban and rural areas, but via different mechanisms [9, pp. 72-73] that will require separate policy responses to be addressed.

Land use differences can also result in challenges that vary from rural to urban areas. For example,* Culex* mosquitoes, the primary vector for West Nile Virus, find the combination of vegetation and the drought/flooding cycle in rural areas an appealing habitat [10, p. 14], whereas, the* Aedes aegypti* mosquito, the primary vector for Dengue, Chikungunya, and Zika virus [11], prefers to live around residences in urban areas [12]. Both types of mosquitoes and the diseases they carry are highly responsive to changes in the climate. However, the vector control methods used to control them differ greatly.

Additionally, rural areas face different infrastructure challenges from urban areas, due to the low population density over large swathes of land. For example, during an extreme precipitation event, rural water basins can flood faster than urban areas; however, these areas often rely on low water crossings rather than bridges to cross water bodies, placing motorists at risk during flooding events. Furthermore, fewer alternate routes and transportation options are available in rural versus urban areas; and, rural communities often experience slower emergency response times than their urban neighbors [13, pp. 106-107]. After an event ends, it also can take longer to restore utilities to rural communities than to metropolitan regions [4].

Rural demographics include a disproportionate percentage of populations that have been shown to be vulnerable to climatic events. Rural populations tend to be older, less affluent, less well educated, and suffering from higher levels of unemployment than urban residents [5, 6]. Of these demographic characteristics, elderly populations and families living in poverty have also been identified as populations who are particularly vulnerable to climatic events ([14–30]; [13, p. 108]). Population health status as a whole is often lower in rural areas than in urban areas, particularly in relation to chronic disease, in spite of rural residents' spending a higher proportion of their income on medical care than their urban counterparts [31]. Furthermore, the fragility of the rural built and emergency response infrastructure exacerbates the vulnerability of groups such as the elderly, families living in poverty, people of color, and populations with limited English proficiency, who have a higher likelihood of living in isolated rural areas [32, p. 252].

These demographic characteristics are often closely intertwined, due to a confluence of social, political, and economic structures collectively referred to as the social determinants of health. Rural populations representing a combination of high-risk social determinants of health (such as elderly populations living in poverty in a flood-prone area) can experience an amplification of the three elements comprising climate change vulnerability [32, p. 249]—increased exposure, increased sensitivity, and reduced adaptive capacity—and should be prioritized for investment in both adaptation and emergency response interventions.

Finally, rural economies are more vulnerable to the negative effects of climate change because they rely on a combination of agriculture and heritage industries such as mining and heavy manufacturing [4, p. 335]. Many agricultural products are already facing climate-related challenges, such as shifting growing seasons and changing precipitation patterns, which will increase as the climate continues to change [33]. Agriculture and industry combined represented over 30% of US greenhouse gas (GHG) emissions in 2014, with the electric power industry (many of whose installations are located in rural areas) contributing an additional 30% [34]. With 60% of total US GHG emissions sourced from the economic engines of rural areas, these communities are particularly vulnerable to the negative economic consequences of GHG reduction policies. Additionally, agriculture is sensitive to changes in seasonal weather patterns [35, 36]. On the other hand, mitigation activities such as reforestation and large-scale renewable energy installations may reinvigorate some rural economies and hurt others [4, p. 340].

In spite of comprising the large majority of the nation's landmass and a sizeable minority of the country's population, less data is available quantifying the vulnerability of rural areas to climate-related environmental hazards than for urban areas, due to the challenges of developing robust statistical models in areas with low densities of both people and environmental sensors such as weather stations [37].

Local health departments are key players in protecting their communities from the negative health effects of climate change, both as participants in hazard mitigation planning and response [38] and as leaders in ongoing efforts to build resilience among vulnerable populations [39]. However, to date, fewer data sources and public health intervention opportunities are available for rural local health departments than for their urban counterparts [40]. The Third US National Climate Assessment identifies vulnerability assessments in rural areas as a key research gap [4, p. 340]. This need is particularly evident in the shortage of indicators measuring the health effects of specific climatic hazards in rural areas.

### 1.1. Environmental Public Health Indicators

Environmental public health indicators are a key component of vulnerability assessments. They are the building blocks for assessing a population's exposure and underlying vulnerabilities to environmentally related health threats such as climate change. They can also be used to track the success of policy and programmatic interventions aimed at reducing vulnerability. However, existing indicators often do not distinguish between rural and urban geographic areas—either aggregating to a larger geographic area (such as the state or county level) or not providing information for sparsely populated areas.

The National Environmental Public Health Tracking (EPHT) Program, hosted by the US Centers for Disease Control and Prevention (CDC), shares key vulnerability and health outcome environmental public health indicators at the national, state, and county levels for a wide array of environmental hazards, including climate change [41]. In 2014, Kentucky received a grant from the CDC to develop a statewide tracking program, EnviroHealthLink, based on the national framework [42]. The Green River District Health Department (GRDHD) conducted the project reviewed in this article under a grant from EnviroHealthLink. The project's goal was to reduce vulnerability to the negative health effects of climate change in a predominately rural region of Western Kentucky. Recognizing the resource and capacity constraints at a rural local health department, we set the objective of using existing online tools and datasets to bring an evidence-base to GRDHD activities relevant to climate change. To this end, we tailored EnviroHealthLink for use at the local level. Using the National EPHT online portal as a foundational resource for gathering datasets, we developed climate change environmental public health indicators relevant to the needs of a rural district. Our second objective was to identify opportunities for integrating the indicators into ongoing public health efforts, such as the community health assessment process and existing health surveillance programs. Our third objective was to identify opportunities for collaboration with partnering agencies, to ensure that vulnerable populations are prioritized in all resilience activities.

## 2. Materials and Methods

### 2.1. Applying the National Environmental Public Health Tracking Framework at the Local Level

**Figure 1:**
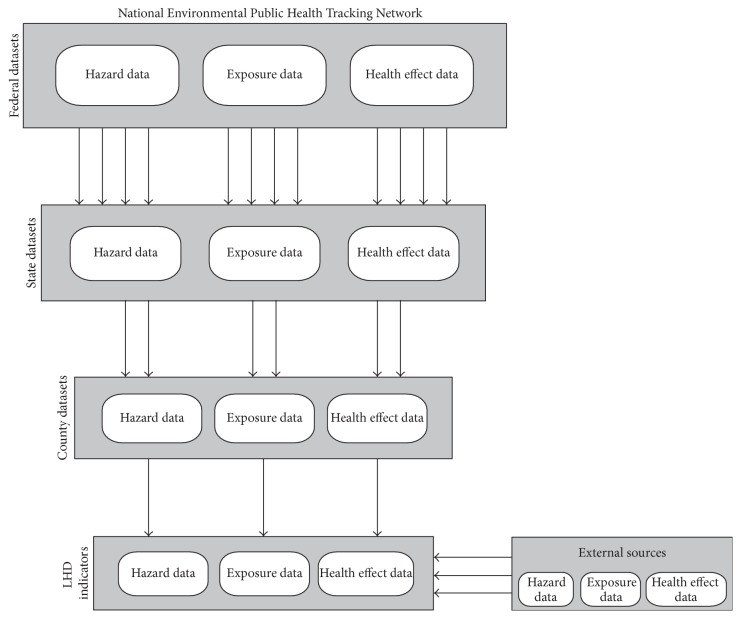
National EPHT Dataset Availability by Spatial Scale.* Note*. Adapted from the CDC's Environmental Public Health Tracking Program Conceptual Model, available at http://www.cdc.gov/nceh/tracking/pdfs/diagram.pdf.

The CDC's National EPHT Network was designed to compile data on environmental hazards, human exposure, and health effects into a single network of standardized databases. This framework enables researchers to evaluate relationships between datasets that historically have been difficult to review side by side [41]. Data is currently available via an online portal at three spatial scales: federal, state, and county. While the majority of datasets are available at the federal level, the CDC funds 25 states (including Kentucky) and 1 city to assist in compiling and making public the smaller spatial scales [43]. Fewer datasets are available at the county level than at the state and federal levels (Figure 1), presenting a challenge to local health departments using the National EPHT Network to support evidence-based policies and programs. The framework presented in this article (Figure 1) recognizes the need to supplement National EPHT datasets with external sources for two purposes: (a) to assess a community's vulnerability to the health effects of climate change and (b) to establish metrics for evaluating the success of local climate and health policies.

### 2.2. Selecting Environmental Hazards for Indicator Development

**Figure 2:**
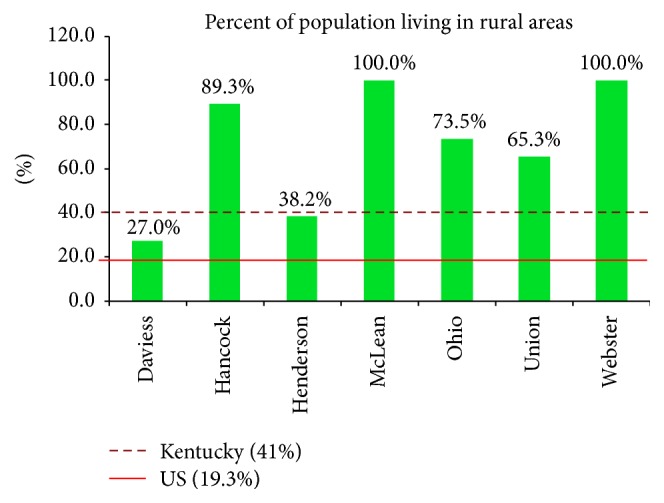
Percentage population classified as rural, by county (KY/GRD: 2016, US: 2010).* Sources*. KY/GRD (2016): County Health Rankings, http://www.countyhealthrankings.org. US (2010): US Census Bureau, Population Division, http://www.census.gov.

The Green River District (GRD) is comprised of seven counties in Western Kentucky: Daviess, Hancock, Henderson, McLean, Ohio, Webster, and Union. It is a predominantly rural area, with two major population centers, Owensboro and Henderson. Total population in 2014 fell just shy of 215,300 residents [44]. All seven counties in the district have a higher percentage of rural residents than the national average (19.3%); and five of the seven exceed the Kentucky average of 41% (Figure 2). The diversity in land use represented across the district, particularly comparing the two more urban counties (Daviess and Henderson) with the five fully rural counties, presents an opportunity to assess the unique challenges and opportunities facing rural health districts engaged in protecting their populations from the negative health effects of climate change.

We used a three-step review process adapted from the CDC's Building Resilience Against Climate Effects (BRACE) framework to identify which climate-related hazards should be developed into environmental public health indicators for the GRDHD. The BRACE framework is a five-step approach to informing public health adaptation efforts at the state and local levels. The five steps are as follows: (1) Anticipating Climate Impacts and Assessing Vulnerabilities; (2) Projecting the Disease Burden; (3) Assessing Public Health Interventions; (4) Developing and Implementing a Climate and Health Adaptation Plan; and (5) Evaluating Impact and Improving Quality of Activities [45]. The GRDHD project completed a portion of Step  (1): Anticipating Climate Impacts and Assessing Vulnerabilities.

First, we reviewed the Third National Climate Assessment [46] and the associated scientific assessment of the impacts of climate change on human health [40] to develop a short list of climate-related environmental hazards with a history and/or projected future of risk to human health in the Southeastern US. We then gathered evidence at a more granular level [47–50] to identify which hazards were associated with the most negative health outcomes and highest economic burdens in rural Western Kentucky. Finally, we consulted with climatologists, emergency management officials, and other subject matter experts at the local and state levels to validate our selection of extreme heat, drought, and flooding as the leading climate-related hazards for the region.

#### 2.2.1. Extreme Heat

As temperatures rise due to the buildup of GHG in the Earth's atmosphere, regions such as Western Kentucky can expect to see increases in both average annual temperatures and the number of extreme heat days each year. By 2050, the average annual temperature in the Green River District is projected to increase from close to 4°F (under a low emissions scenario) to 5°F (under a high emission scenario) [47, p. 15]. By 2020–2039, Kentucky as a whole is projected to experience up to 23 days per year with temperatures exceeding 95°F. From 2040 to 2059, up to 44 days per year could exceed 95°F. Western Kentucky is projected to warm more than other regions in the commonwealth [51, pp. 44-45].

Given these projections and in consultation with the state climatologist, we defined extreme heat exposure as three or more days with maximum temperatures greater than or equal to 95 degrees.

Exposure to extreme heat can inhibit the body's natural ability to regulate its internal temperature. It can also exacerbate cardiovascular, respiratory, and cerebrovascular diseases [52, p. 46]. Heat combined with humidity and extended exposure to extreme heat alone can be debilitating, reducing an individual's ability to concentrate and leading to fatigue ([52, p. 46]; [53, 54]). From a mental health perspective, extreme heat has been linked with increases in aggressive behavior and hospital admittances for psychiatric conditions [53, 55, 56]. The combination of heat and humidity may also correlate with increases in suicide rates, although current findings are not conclusive [53, 54].

Population vulnerability to extreme heat includes individuals on either end of the age spectrum. Both children and the elderly have a limited capacity to regulate their internal temperature [14]. Both groups are also likely to rely on others to keep them safe during heat events [15–19, 57]. Families living in poverty are at risk, because they may not have sufficient access to heat-related adaptations such as weatherized buildings and affordable air conditioning [15, 17, 18, 20–22]. Non-Hispanic Black populations are often at higher risk than the general population because of a combination of health status, socioeconomic status, and environmental justice concerns [15, 17, 18, 21, 23–25, 58]. Homeless populations may combine increased exposure to heat and cold with other risk factors such as social isolation, psychiatric illness, and multiple chronic diseases [59]. Outdoor workers are at increased risk of negative health outcomes during extreme heat events, due to increased exposure to elevated temperatures during the heat of the day [19, 60, 61].

Preexisting chronic health conditions can also place an individual at higher risk of negative health outcomes during an extreme heat event. For example, obese individuals are more sensitive to high ambient temperatures [3, p. 34]. Similarly, exposure to heat can exacerbate conditions such as diabetes, cardiovascular disease, asthma, and cerebrovascular disease [52, p. 46].

#### 2.2.2. Drought

Kentucky has experienced a trend of increasing drought conditions since the 1950s [62]. As temperatures warm and precipitation patterns become less reliable, these events are likely to increase in frequency ([47, pp. 13-14 and Figure 4]; [63, 64]). According to Climate Central, the severity of summertime droughts in Kentucky is expected to double by 2050 [65].

We defined exposure to drought as a severe (D2), extreme (D3), or exceptional (D4) drought declaration by the US Drought Monitor.

It can be difficult to quantify the direct health effects of drought, because they tend to result from a complex interplay of socioeconomic variables (such as loss of livelihood); local environmental characteristics (such as land use patterns); and interactions between the drought and other related natural events (such as the prevalence of wildfires or the duration of a heat wave) [66, 67]. As a result, we did not develop direct health outcomes for this hazard. However, exposure to fine particulate matter (in the form of dust) could be used as a proxy for the health effects of drought associated with poor air quality.

The population vulnerability indicators associated with drought overlap with extreme heat, in part, because the two hazards can occur concurrently. Children and the elderly are particularly vulnerable to the effects that drought can have on air quality and water quality [23, 24, 26–28, 68–72]. Similarly, individuals with diabetes, chronic lower respiratory disease (CLRD), and asthma are at heightened risk due to their sensitivity to poor air quality [26–28, 73]. Finally, rural areas have been associated with more severe mental health concerns (such as anxiety, depression, and posttraumatic stress disorder (PTSD)) during droughts than urban areas, due to the strong economic relationship between precipitation and agricultural yields [66, 74–77]. Social networks in rural areas can be disrupted when populations relocate during a drought to seek alternative employment [53, 54, 66, 74–77]. This vulnerability is compounded by the difficulty of accessing mental health services in many rural communities ([4, p. 339]; [54]).

#### 2.2.3. Flooding

Major disasters were declared in Kentucky due to flooding and/or severe storms 29 times from 2000–2015, damaging 6,000 homes—many of which belonged to low-income families. The number of declarations over that fifteen-year period is almost equivalent to the previous forty years combined [78]. Western Kentucky has historically experienced 55 thunderstorm days per year [79], with the 30-year average of total annual precipitation slowly trending upwards starting in the 1960s and a more marked rise (particularly during spring months) from the 1990s onward [80]. Average annual precipitation in the GRD is projected to increase 2% under a low emissions scenario and up to 6% under a high emissions scenario [47, p. 16].

We defined exposure to flooding as the number of days annually with precipitation over 2 inches, as reported by weather stations.

The health effects of flooding range from drowning-related injury and death [24, 81–86] to gastrointestinal illnesses [24, 85] and mental health concerns [24, 54, 85, 86]. Other flooding-related morbidities and mortalities are more difficult to track, because flooding may not be listed as a primary cause in the diagnosis or on the death certificate [13, p. 114]. However, it can be extrapolated in some cases. For example, poor indoor air quality arising from dampness and mold in the home is currently associated with 8%–20% of respiratory infections in the US (such as acute bronchitis) and with exacerbating 4.6 million cases of asthma [87, 88]. Flooding events can also result in power outages, which have been associated with carbon monoxide poisoning caused by using combustion appliances indoors [89, 90].

Similar to extreme heat and drought, children and the elderly are particularly vulnerable to negative health outcomes after exposure to flooding. Both groups are more vulnerable than the population as a whole to flooding-related injuries and illnesses [24, 25]. Children are particularly vulnerable if they are separated from their caregivers [91–93]. Both populations are also particularly susceptible to the negative mental health effects of flooding ([13, p. 108]; [54]) associated with the loss of property and loved ones, economic hardship, and dislocation. These health outcomes include anxiety, PTSD, aggression in children, and suicidal tendencies [24, 53, 85, 86]. The dangers associated with medical disruptions also led to the inclusion of long-term care facilities [24, 29, 30, 94–99] and diabetes [100, 101] on the list of vulnerability indicators for the GRD.

### 2.3. Populating Baseline Environmental Public Health Indicators at the Local Level

To assess the Green River District's relative vulnerability to each hazard, we compiled environmental exposure, human health outcome, population vulnerability, and environmental vulnerability indicators drawn from the literature review for each hazard.  Table 1 illustrates that while a number of key indicators were available on the National EPHT Network portal (most notably, environmental exposure indicators) external sources were required to perform a comprehensive assessment at the local level. For example, a number of studies have established a correlation between extreme heat events and heat-related morbidity and/or mortality by comparing the dates of extreme heat events with the dates of heat-related morbidity and mortality data [102–105]. While the National EPHT portal provides heat-related mortality data for Kentucky during summer months from 1999 to 2014, it does not provide data at the county level. Furthermore, the datasets are annualized to protect privacy [106]. However, county-level temperature data from 2000 to 2012 (also obtained from the National EPHT portal) shows geographic variation across the district [106]. This variation is likely even more pronounced across the commonwealth. In order to compare morbidity and mortality data with the actual dates meeting the definition of an extreme heat event, we gathered heat exposure data from the Kentucky Climate Center and heat-related morbidity and mortality data from the Kentucky Department for Public Health. The Kentucky Injury Prevention and Research Center at the University of Kentucky retrieved the health data from the Kentucky Inpatient Hospitalization and Outpatient Services Claims Files at the Kentucky Cabinet for Health and Family Services, Office of Health Policy, and from the Kentucky Death Certificates Files at the Kentucky Department for Public Health, Cabinet for Health and Family Services. It received Institutional Review Board approval from the Kentucky Cabinet for Health and Family Services.

Similarly, while robust, the population and environmental vulnerability data available on the National EPHT portal are not as comprehensive as the list of correlations in the public health literature. It was therefore necessary to gather a number of local level datasets from sources such as the US Census, CDC databases outside of the EPHT program, and the Kentucky Department for Public Health (Table 1). Attempts to gather local level mental health data were unsuccessful. Comprehensive mental health data is lacking for most of the identified region.

The project's short timeline, limited funding, and capacity constraints precluded the use of statistical analysis to identify the subset of indicators that were most relevant to each county or the region as a whole. The project report recommends filling this gap under the next round of funding. Given this constraint, which is not uncommon at local health departments, we directly compared each local indicator with parallel Kentucky and US datasets, if available, to establish a rough understanding of which indicators might prove to be outliers using more sophisticated methods of analysis. Five indicators were also compared with federal standards: exposure to air pollution (compared with US EPA 2012 annual standard for fine particulate matter concentrations [107]), obesity (compared with Healthy People 2020 Goal NWS-9 [108]), heart disease mortality (compared with Healthy People 2020 Goal HDS-2 [109]), asthma hospitalizations (compared with Healthy People 2020 Goal RD-2.2 [110]), and cerebrovascular deaths (compared with Healthy People 2020 Goal HDS-3 [109]). Indicators were defined as high vulnerability if they fell short of both Kentucky and US indicators. They were defined as moderate if they fell in between the larger-scale indicators. And they were defined as low if they represented an improvement over both the Kentucky and US indicators.

## 3. Results

### 3.1. Rural Vulnerabilities of the Green River District

The Green River District demonstrates high risk for two of the key vulnerable populations characteristic of rural areas, as identified in the scientific literature: the elderly and families living in poverty (Table 2). All seven counties in the district host a slightly higher proportion of elderly populations (aged 65+) than the national average or the commonwealth of Kentucky. Every county except for Hancock also exceeds the national proportion of populations living in poverty. However, only Ohio County also exceeds both the national and Kentucky average.

Of the chronic diseases that can increase vulnerability to the negative health effects of extreme heat, drought, and flooding, many of the counties in the Green River District report higher levels of diabetes, heart disease, CLRD, asthma, and cerebrovascular disease than both Kentucky and the US as a whole (Table 2). Due to a lack of baseline data for the GRD region, comparisons between county mental health status and state and national standards are not shown.

### 3.2. Extreme Heat Vulnerability

**Figure 3:**
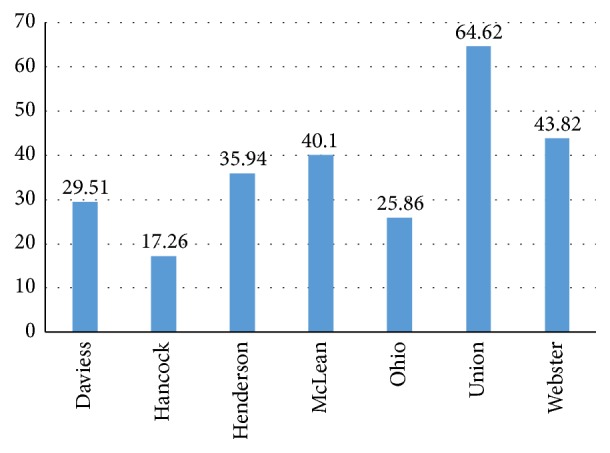
Heat-related emergency department visits per 10,000 by county (2008–2012).* Source*. Kentucky Inpatient Hospitalization and Outpatient Services Claims Files at the Kentucky Cabinet for Health and Family Services, Office of Health Policy. Retrieved by the Kentucky Injury Prevention and Research Center at the University of Kentucky.

Table 3 displays the dates from 2000 to 2012 when one or more county in the district met the project's extreme heat exposure definition. Interestingly, two of the most rural counties in the district (Union and Webster) experienced the highest number of extreme heat events over this time period. Union County also displayed the highest rate of heat-related emergency department visits in the district from 2008 to 2012 (Figure 3).

The elderly were identified in all seven counties as a population with high vulnerability. Children were similarly identified for five counties, excluding Union and Webster. Homeless populations were identified as highly vulnerable in Daviess, the most urban county in the district. In contrast, outdoor workers were identified as a high vulnerability population in the five particularly rural counties—Hancock, McLean, Ohio, Union, and Webster. Obesity and/or diabetes and heart disease were also identified as indicators of high vulnerability to extreme heat in the same five counties (Table 2).

### 3.3. Drought Vulnerability

**Figure 4:**
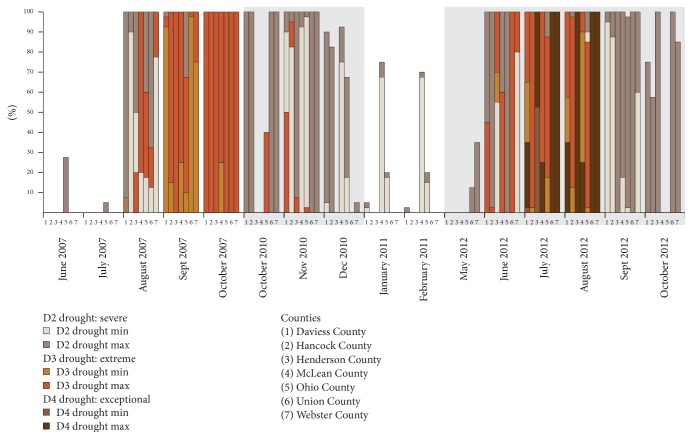
Percent population impacted by drought in Green River District counties (2007–2012).* Source*. US Drought Monitor, http://droughtmonitor.unl.edu/Home.aspx.

From 2007 to 2012, the district experienced three widespread droughts meeting the project's definition: August–October 2007, October–December 2010, and May–October 2012 (Figure 4). Furthermore, the most intensive drought, July-August 2012, coincided with 33 days of extreme heat exposure (Table 3). During those two months, exceptional drought (D4) was declared in five of the seven counties, demonstrating the complex interplay between climate-related hazards such as extreme heat and drought.

The average concentration of PM_2.5_ in the Green River District (14.1 PM_2.5 _*μ*g/m^3^) was slightly higher than the Kentucky average (13.5 PM_2.5 _*μ*g/m^3^) in 2011 and significantly higher than the national average of the same year (11.1 PM_2.5 _*μ*g/m^3^ nationally) [111].

The demographic picture for high vulnerability drought indicators in the Green River District aligns with extreme heat regarding children and the elderly. Daviess County again stands out from the rest of the region by identifying a subpopulation as highly vulnerable that is not picked up in the other six counties: individuals requiring mental health services. Diabetes is identified as a high vulnerability indicator in the rest of the region, along with CLRD and/or asthma in Hancock, Henderson, and Webster Counties (Table 2).

### 3.4. Flood Vulnerability

**Figure 5:**
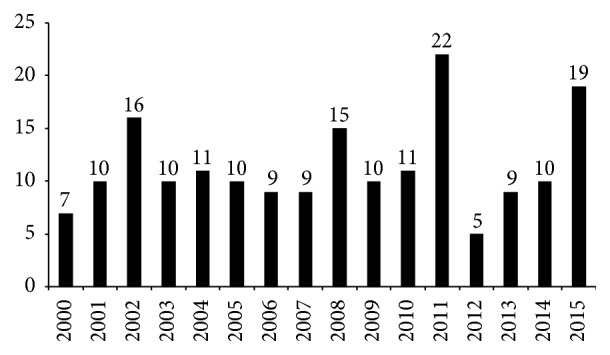
Number of flooding events in Green River District by year (2000–2015).* Source*. NOAA National Climatic Data Center Storm Events Database, http://www.ncdc.noaa.gov/stormevents.

Flooding and severe thunderstorms accounted for 42% of natural hazard events and 92% of related property damage in the Green River District from 2010 to 2015 [48, Figures 4-1 and 4-2] with serious flooding occurring, on average, every two years [48, p. 109]. The entire GRD averaged at least 15 days of high water flow annually from 2000 to 2009, with some areas experiencing 23 days or more [112].  Figure 5 displays the number of flooding events in the district reported in the NOAA National Climatic Data Center Storm Events Database from 2000 to 2015. During the same period, the direct economic effects of flooding in the area reached close to $40 million in property damage and $6 million in crop damage [50].

Residents of the GRD are highly vulnerable to flooding, in part, due to development in known floodplains. The FEMA floodplain was listed as a high vulnerability indicator for all counties in the district except for Webster County, because they exceed the Kentucky (5.4%) percentage of populations living in the 100-year floodplain (Table 2). Three counties—Daviess (13.6%), Hancock (14.7%), and McLean (12.8%)—demonstrate more than double the average statewide exposure [106]. From a geographic perspective, every county except Webster also greatly exceeds the percentage of Kentucky (9.8%) land located in a floodplain, with close to one-half of the land in Henderson (43.2%) and McLean (45.3%) counties located in vulnerable areas [106]. In spite of the relatively high percentage of residents in the district living in a floodplain, less than 2% hold current flood insurance policies [113], further increasing their vulnerability to the economic effects of flooding.

The district experienced 7 unintentional drowning-related mortalities from 2008 to 2015 [114]. From 2008 to 2013, emergency department visits in the Green River District related to carbon monoxide poisoning ranged from 11 in Union and Webster Counties to 36 in Daviess County [115].

Similar to drought, mental health concerns were only identified as a high vulnerability indicator for flooding exposure in the more urban Daviess County. Patients in long-term care facilities were identified as an indicator with high vulnerability in Daviess, Hancock, McLean, and Ohio counties. And diabetes was identified as a high vulnerability indicator in five of the seven counties: Hancock, Henderson, McLean, Ohio, and Webster (Table 2).

## 4. Discussion

Comparing the baseline climate change environmental public health indicators for the Green River District, Daviess County's more urban status distinguished it from the other six counties, particularly in terms of the need to develop educational messaging and services for homeless populations. Baseline indicators for the more rural counties, on the other hand, included an emphasis on outdoor workers and populations with existing chronic conditions such as diabetes, heart disease, and asthma. These differences indicate that climate and health policies and interventions in the Green River District must distinguish between the needs of rural and urban populations in the seven-county region.

While current policies and programs in the GRDHD do not explicitly address climate change, three areas of current engagement are particularly relevant to the study results described above: epidemiology, public health preparedness, and community health assessment.

Initial research highlighted mental health awareness as a major weakness in the GRD region. Due to the lack of focus on mental health within current programs and data collection, GRDHD will be considering the status of mental health conditions, the rural impact on mental health services and access, and environmental influences on mental health for future grants and programs.

The GRDHD epidemiology program monitors the health status of the community and investigates disease clusters and outbreaks. A passive surveillance system is already in place, tracking infectious diseases reported by health care providers and other community members. Several reportable conditions are relevant to climate and health surveillance, such as mosquito-borne diseases (West Nile Virus, Zika Virus, etc.) and waterborne disease outbreaks [116]. The epidemiology department also provides morbidity and mortality surveillance to the state health department during community outbreaks and disasters. Based on the results of this study, the GRDHD plans on expanding both the surveillance and reporting arms of the epidemiology department to track environmental exposure indicators for all three climate hazards and reportable health outcomes for extreme heat and flooding (Table 4). Future work may include correlating health outcome data with the dates when extreme heat or flooding events occurred, in order to validate or change the thresholds established during this project to trigger heat-, drought-, and flooding-related surveillance activities, public education campaigns, and interventions such as opening cooling centers and emergency shelters.

The health department supports local emergency preparedness agencies to safeguard the health of vulnerable populations during and after both natural and human-caused disasters. Much of this work involves public communication—explaining the health risks associated with an event and ways for individuals to protect themselves and their families. The health department plans to use the results of this study to tailor heat, drought, and flooding emergency response activities to the highest risk populations in each county.

An active surveillance program will also be developed to collect health data on clients of cooling centers and emergency shelters. Example datasets to be collected may include Injuries, Dermatologic, Gastrointestinal Illness, Pregnancy, Respiratory Illness, Pain, Dehydration, Fever, Exacerbation of Chronic Disease, and Mental Health. Furthermore, the health department will work with the Green River Area Development District to incorporate the results of the study into the upcoming revision of the regional hazard mitigation plan.

Finally, the study results have been distributed as an addendum to the 2015 Green River Community Health Assessment (CHA),* Climate and Health Addendum to 2015 Green River Community Health Assessment* (http://healthdepartment.org/community-health/community-health-plans/). The report's focus on vulnerable populations aligns with the goals of the CHA. Many of the populations identified by the climate and health scientific literature as vulnerable to extreme heat, drought, and/or flooding have also been identified in the CHA as requiring special consideration under other health-promoting programs. For example, the 2015 CHA tracks the percentage of children, elderly, non-Hispanic Blacks, and populations living in poverty for each county in the Green River District. Additionally, mental health concerns and language barriers are highlighted as areas in need of improvement to advance community health. The assessment also highlights three Kentucky Health Now Goals that overlap with the climate and health indicators listed in Table 1: obesity, cardiovascular disease, and mental health [117]. Local communities utilize the health assessment to establish priorities and develop strategic planning efforts to improve the health and resiliency of their community. The most recent CHA, published in 2015, focuses on reducing substance abuse, reducing obesity, reducing teen pregnancy, and improving access to health care and mental health services. The climate and health addendum will facilitate conversations about the links between climate change and environmental health during the next planning process, due to begin in 2017.

### 4.1. Strengths and Limitations

The project reviewed in this article addressed a gap in both climate and health research and practice, namely, the shortage of vulnerability assessments in rural areas [4, p. 340]. Its focus on developing EPH indicators at the local level is also rare. Two examples at that spatial scale, Reid et al. 2009 [118] and Prudent et al. 2016 [119], developed composite climate change vulnerability indices based on a core set of indicators. Importantly, these indices were mapped to subcounty spatial scales to help local authorities and partners pinpoint the locations within counties where multiple vulnerabilities converge. This information can be used to target investment in adaptation and emergency response efforts.

Due to schedule, resource, and capacity constraints, the phase of the project reviewed by this article did not include the mapping, spatial analysis, and statistical validation performed by Reid et al. and Prudent et al. That additional step is necessary to identify the subgroups and specific locations at highest risk of negative health effects after exposure to extreme heat, drought, and/or flooding. However, additional quantitative analysis and validation were identified in the project report as important objectives pending future funding opportunities.

The final product may bear resemblance to a localized version of the output of several grantees under the CDC's Climate Ready States and Cities program, which have followed the BRACE framework for climate and health adaptation. For example, Arizona has developed maps of social vulnerability, impervious surface, weather-related, and county warning area indicators to identify the locations in the state that are most vulnerable to negative health outcomes during extreme heat events (http://www.azdhs.gov/preparedness/epidemiology-disease-control/extreme-weather/index.php#heat-maps). Illinois has developed geospatial indices at the county level for social vulnerability, flooding vulnerability, and the effects of ozone on asthma vulnerability (https://braceillinois.uic.edu/). And the Minnesota Department of Health has included climate-related environmental health and human health indicators (such as air quality, asthma, chronic obstructive pulmonary disease, diabetes, heat-related illness, and Lyme disease) in its overall data access portal (https://apps.health.state.mn.us/mndata/home).

## 5. Conclusions

Climate change represents a significant and growing threat to population health. Rural areas, such as the Green River District in Kentucky, face unique challenges that are often overlooked by climate and health policies and programs. This project addressed that gap by adapting the National Environmental Public Health Tracking Network's framework for climate and health indicators to a seven-county, rural health department in Western Kentucky.

A review of the public health literature identified three primary climate-related environmental public health hazards for the region (extreme heat, drought, and flooding) and a suite of related exposure, health outcome, population vulnerability, and environmental vulnerability indicators. Indicators that performed more poorly at the county level than at the state and national levels were defined as “high vulnerability.” The most urban county, Daviess, illustrated the urban/rural divide in relation to vulnerable populations—for example, identifying homeless populations and populations with mental illness as highly vulnerable groups in comparison with outdoor workers and populations with existing chronic conditions, as seen in the more rural counties.

The health department plans to use the results of this study to enhance three key areas of their existing services: epidemiology, public health preparedness, and community health assessment.

## Figures and Tables

**Table 1:** Availability of Green River District climate and health indicator datasets from the EPHT Network, external sources.

Indicator category	Data available from EPHT Network *(notes)*	Data from external source *(source)*
Environmental exposure	Exposure to heat waves^h^ Exposure to heavy precipitation events^f^ Exposure to air pollution^d^	Exposure to heat waves^h^ *(Kentucky Climate Center)* Exposure to drought^d^ *(US Drought Monitor)* Exposure to air pollution^d^ *(CDC WONDER)* Exposure to heavy precipitation events^f^ *(Kentucky Climate Center, National Weather Service)*

Human health outcome	Heat-related mortality during summer months^h^ *(annualized data; not available at county level)*	Heat-related morbidity and mortality during extreme heat events^h^ *(Kentucky Cabinet for Health and Family Services, Office of Health Policy; Kentucky Department for Public Health, Cabinet for Health and Family Services)* Unintentional flooding-related mortality during flooding events^f^ *(Kentucky Department for Public Health, Cabinet for Health and Family Services, CDC WONDER)* Unintentional flooding-related morbidity during flooding events^f^ *(Kentucky Cabinet for Health and Family Services, Office of Health Policy; Kentucky Department for Public Health, Cabinet for Health and Family Services)*

Population vulnerability	Asthma^h,d^ Diabetes^h,d,f^ Heart disease^h^ *(not available at county level)* Obesity^h^ *(not available at county level)* Poverty^h,f^ *(not available at county level)*	Children^h,d,f^, elderly^h,d,f^, population living in poverty^h,f^, non-Hispanic Blacks^h,d,f^, outdoor workers^h^, population with limited English proficiency^f^, ambulatory difficulty^f^*(US Census)* Homeless^h^ *(Kentucky Housing Corporation, US Housing and Urban Development)* Long-term Care^f^ *(Kentucky Cabinet for Health and Family Services Office of Health Policy)* Chronic lower respiratory Disease^d^ *(CDC Community Health Status Indicators)* Diabetes^h,d,f^ *(Kentucky Behavioral Risk Factor Surveillance System)* Heart disease^h^, cerebrovascular disease^h^ *(CDC Interactive Atlas of Heart Disease and Stroke)* Mental health^d,f^ *(Kentucky Safety and Prevention Alignment Network)* Obesity^h^ *(CDC Behavioral Risk Factor Surveillance System)*

Environmental vulnerability	100-year floodplain^f^ Carbon monoxide poisoning^h,f^ *(KY data currently not available via EPHT portal)*	Air conditioning access^h^ *(Energy Information Administration Residential Energy Consumption Survey)* Carbon monoxide poisoning^h,f^ *(Kentucky Cabinet for Health and Family Services)* Stressed housing^h,d,f^ *(CDC Community Health Status Indicators)*

*Notes*.

^h^Heat indicator.

^d^Drought indicator.

^f^Flooding indicator.

**Table 2:** High vulnerability by climate hazard in Green River District counties.

County	Indicators with high vulnerability	Extreme heat	Drought	Flooding
Daviess	Children	✓	✓	✓
	Elderly	✓	✓	✓
	Homeless	✓		
	Mental health		✓	✓
	Long-term care			✓
	FEMA floodplain			✓

Hancock	Children	✓	✓	✓
	Elderly	✓	✓	✓
	Outdoor workers	✓		
	Diabetes	✓	✓	✓
	Heart disease	✓		
	CLRD		✓	
	Long-term care			✓
	FEMA floodplain			✓

Henderson	Children	✓	✓	✓
	Elderly	✓	✓	✓
	Diabetes	✓	✓	✓
	CLRD		✓	
	Asthma	✓	✓	
	Cerebrovascular disease	✓		
	FEMA floodplain			✓
	Stressed housing	✓	✓	✓

McLean	Children	✓	✓	✓
	Elderly	✓	✓	✓
	Outdoor workers	✓		
	Diabetes	✓	✓	✓
	Heart disease	✓		
	Cerebrovascular disease	✓		
	Long-term care			✓
	FEMA floodplain			✓

Ohio	Children	✓	✓	✓
	Elderly	✓	✓	✓
	Poverty	✓	✓	✓
	Outdoor workers	✓		
	Diabetes	✓	✓	✓
	Heart disease	✓		
	Long-term care			✓
	FEMA floodplain			✓

Union	Elderly	✓	✓	✓
	Outdoor workers	✓		
	Obesity	✓		
	Heart disease	✓		
	Cerebrovascular disease	✓		
	FEMA floodplain			✓

Webster	Elderly	✓	✓	✓
	Outdoor workers	✓		
	Obesity	✓		
	Diabetes	✓	✓	✓
	Heart disease	✓		
	CLRD		✓	
	Asthma	✓	✓	
	Cerebrovascular disease	✓		

**Table 3:** Extreme heat exposure in Green River District County (2000–2012): days with maximum temperatures greater than or equal to 95 degrees from May to September.

Years	Extreme heat exposure	Counties affected
2000	8/28/00–8/30/00	Union, Webster

2001	No dates met exposure definition	

2002	8/02/02–8/04/02	Union
	9/07/02–9/10/02	Henderson, Union, and Webster

2003	No dates met exposure definition	

2004	No dates met exposure definition	

2005	7/24/05–7/26/05	Union
	8/09/05–8/14/05	Henderson, McLean, Union, and Webster

2006	7/18/06–7/21/06	Union
	7/30/06–8/03/06	Union, Webster
	8/06/06–8/10/06	Union

2007	7/31/07–8/24/07	All counties
	8/27/07–8/29/07^*∗*^	Henderson, McLean, Union, and Webster
	9/02/07–9/05/07^*∗*^	Daviess, Henderson, McLean, Union, and Webster

2008	No dates met exposure definition	

2009	No dates met exposure definition	

2010	7/23/10–7/25/10^*∗*^ 8/01/10–8/04/10 8/08/10–8/15/10 8/19/10–8/22/10 8/31/10–9/02/10 9/19/10–9/23/10	Hancock All counties All counties All counties All counties All counties

2011	8/31/11–9/03/11	All counties

2012	6/18/12–6/21/12	Henderson, McLean, Union, and Webster
	6/23/12–6/25/12	Henderson, McLean, and Webster
	6/27/12–7/10/12	All counties
	7/15/12–8/09/12	All counties
	8/23/12–8/25/12^*∗*^	Daviess, Henderson, McLean, Union, and Webster
	8/28/12–8/31/12^*∗∗*^	All counties

*Notes*.

^*∗*^All other counties reached threshold for 2 days.

^*∗∗*^Hancock County reached one-degree shy-of-threshold on 8/29/12.

*Source*. National Environmental Public Health Tracking Program, http://ephtracking.cdc.gov/.

**Table 4:** Green River District Health Department proposed climate and health surveillance system.

Hazard	Exposure indicator *(surveillance trigger)*	Reportable health outcome
Extreme heat	Max temperature greater than or equal to 95 for a minimum of 3 days *(Heat advisory issued by National Weather Service).*	Number of heat-related deaths (ICD-10: X30); number of heat stress hospitalizations and emergency department visits (ICD-9: 992, E900.0, E900.9) from May to September.

Drought	County declared in D2 (severe), D3 (extreme), or D4 (exceptional) drought by US Drought Monitor *(Same).*	In development.

Flooding	Number of days with precipitation over 2 inches reported by weather stations annually *(Flood warning issued by National Weather Service).*	Number of unintentional drowning-related mortalities (ICD-10: W69, W70, and X38) and flooding-related hospitalizations and emergency department visits (ICD-9: E908.2, E908.9, E910.8, and E910.9).
